# Multiple aplasia cutis congenita type V and fetus papyraceous: a case report and review of the literature 

**DOI:** 10.1186/s13256-021-02662-3

**Published:** 2021-03-04

**Authors:** V. Thadchanamoorthy, Kavinda Dayasiri, M. Thirukumar, N. Thamilvannan, S. H. Chandraratne

**Affiliations:** 1 Faculty of Health Care Sciences, Eastern University, Chenkalady, Sri Lanka; 2 Consultant Pediatrician, Base Hospital, Mahaoya, Sri Lanka; 3 Teaching Hospital Batticaloa, Batticaloa, Sri Lanka; 4 Dermatology, Teaching Hospital, Batticaloa, Sri Lanka

**Keywords:** Aplasia cutis congenital, Consanguinity, Scalp

## Abstract

**Background:**

Aplasia cutis congenita is regarded as congenital focal absence of skin in the newborn, and occurrence of more than three similar skin defects is rare. The etiology is thought to be multifactorial, and precise etiopathogenesis is unknown.

**Case presentation:**

A 13-day-old newborn Sri Lankan Tamil girl was referred to the dermatologic clinic with multiple skin defects at birth. There were six lesions on the body, and two of them had healed during intrauterine period, leaving scars. This was a second twin of her pregnancy. Her first twin fetus had demised before 19 weeks of pregnancy and was confirmed to be fetus papyraceous based on ultrasound-guided fetal assessment. The said child was thoroughly investigated and found to have no other congenital abnormalities. Chromosomal studies yielded normal findings. She was treated with tropical antibacterial ointment, and all lesions resolved spontaneously within 4 weeks, leaving scars. Physiotherapy was commenced to prevent contracture formation, and follow-up was arranged in collaboration with the plastic surgical team.

**Conclusions:**

Aplasia cutis congenita is a rare condition of uncertain etiology, but consanguinity may play a role. This report described a newborn with type V cutis aplasia congenita in whom the diagnosis was confirmed based on clinical features and revision of antenatal history. The management depends on the pattern, extent, location, severity, underlying causes, and associated anomalies.

## Introduction

Aplasia cutis congenita (ACC) is a heterogeneous group of disorders which was first described by Cordon in 1767. ACC is defined as localized or widespread, complete or partial, absence or scarcity of skin at birth, and lesions occur at various depths of the skin, including absence of epidermis, dermis, and occasionally subcutaneous tissues or even bone tissue. The condition is recognized from the disrupted development between 10 and 15 weeks or degeneration of skin in utero [1]. The incidence is about 3 in 10,000 births, and nearly 500 cases have been reported in literature [2]. However, only 2% of aplasia cutis shows multiple defects in excess of three [3], and type V ACC has been reported in approximately 100 newborns to date.

The etiology of ACC is not known [4], but several pathophysiological mechanisms have been suggested as being causative of either disrupted intrauterine skin development or degeneration. These causes include intrauterine infections [5],vascular accidents [6], placental infarcts and thrombosis [7], chromosomal abnormalities and genetic syndromes [8], teratogenic substances such as misoprostol, benzodiazepines, valproic acid cocaine, methotrexate, angiotensin-converting enzyme (ACE) inhibitors and methimazole [9, 10], trauma [11], and ectodermal dysplasia and imperfect neural tube closure [4]. Management of ACC depends on the magnitude of clinical presentation. Small localized lesions may be managed conservatively, whilst larger lesions might need surgical intervention [12]. Complications include infection, thrombosis, and bleeding and warrant prompt and effective management. Complications can be potentially associated with increased mortality. This report describes a 13-day-old newborn with type V ACC and without evidence of underlying etiology or secondary complications.

## Case history

A 13-day-old Sri Lankan Tamil girl was referred to the dermatologic clinic with multiple skin defects noticed at birth. She was first born to healthy non-consanguineous parents. There was no history of miscarriage or stillbirth. Parents were from middle class with extended family support. Mother had rubella vaccine before marriage and periconceptional folic acid following prepregnancy counseling. Mother did not report any maternal infections, smoking or alcohol consumption during pregnancy, especially in first trimester. This was a dichorionic diaminiotic (DCDA) twin pregnancy diagnosed in her first-trimester ultrasound scan at 10 weeks of gestation and also by elevated maternal alpha fetoprotein (200 ng/L) at 14 weeks of gestation. However, subsequent anomalous scan at 18 weeks of gestation following a history of mild vaginal bleeding confirmed only one viable fetus and demise of the second twin fetus (fetus papyraceous). During the context of pregnancy, she was not on any regular medications except multivitamin, calcium, and iron supplements. She did not report having medical illnesses such as diabetes and hypertension during antenatal period. The pregnancy with single live fetus continued with close follow-up by medical team until 38 weeks of gestation without complications. However, oligohydramnios (reduced amniotic fluid index) was detected at 38 weeks of gestation by ultrasound-guided fetal assessment. Subsequently, she was induced with prostaglandin, and the baby was delivered in good condition. Baby’s maturity was term, and Apgar was 10 at 5 minutes. Birth weight of the reported newborn was 2.85 kg, head circumference was 34 cm, length was 54 cm, and abdominal circumference was 34 cm. At birth, baby had a pulse of 138 beats/minute, blood pressure of 65/30mmHg, and temperature of 36 °C. Neonatal examination including hip and palate was normal, but six skin defects were noticed on her skin. Neurological examination revealed conscious-reactivity, spontaneous motor activity, normal neck, trunk, and limb muscle tone, well-developed Moro, grasping, and sucking reflexes and appropriate startle response. Placental calcifications were also noted on macroscopic examination. There was no family history of similar defects. Baby established breast feeding, and Bacillus Calmette–Guérin (BCG) vaccination was administrated within 24 h. The baby was screened for other abnormalities and found to be normal, being observed at mother–baby unit.

**Fig. 1:**
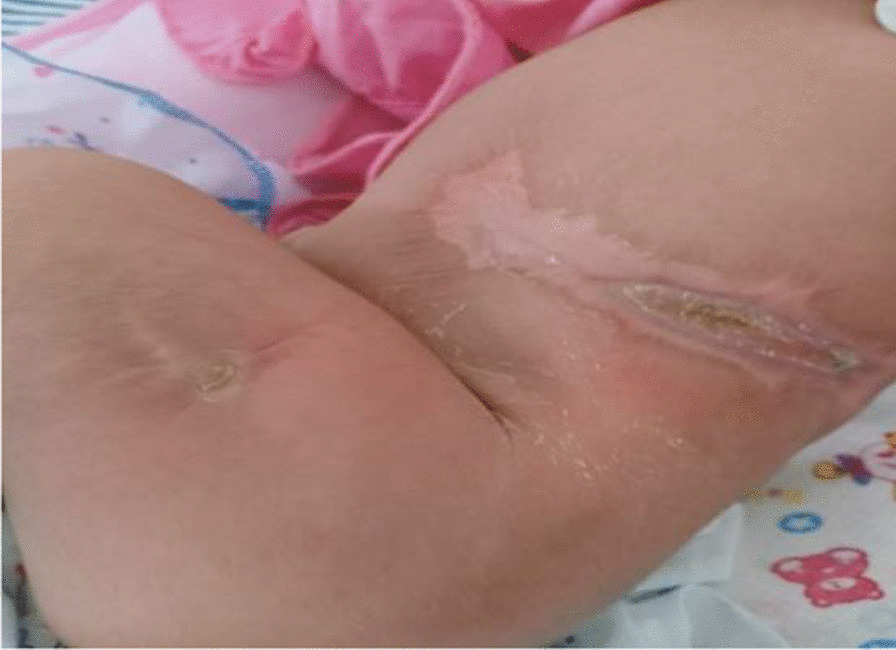
Aplasia cutis distributed over left side of abdomen

**Fig. 2:**
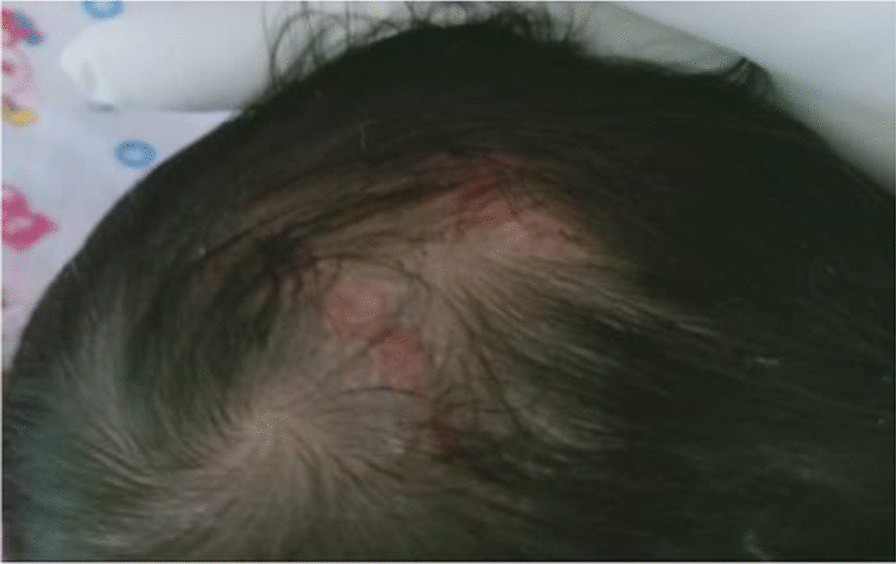
Two skin defects noticed on head

**Fig. 3:**
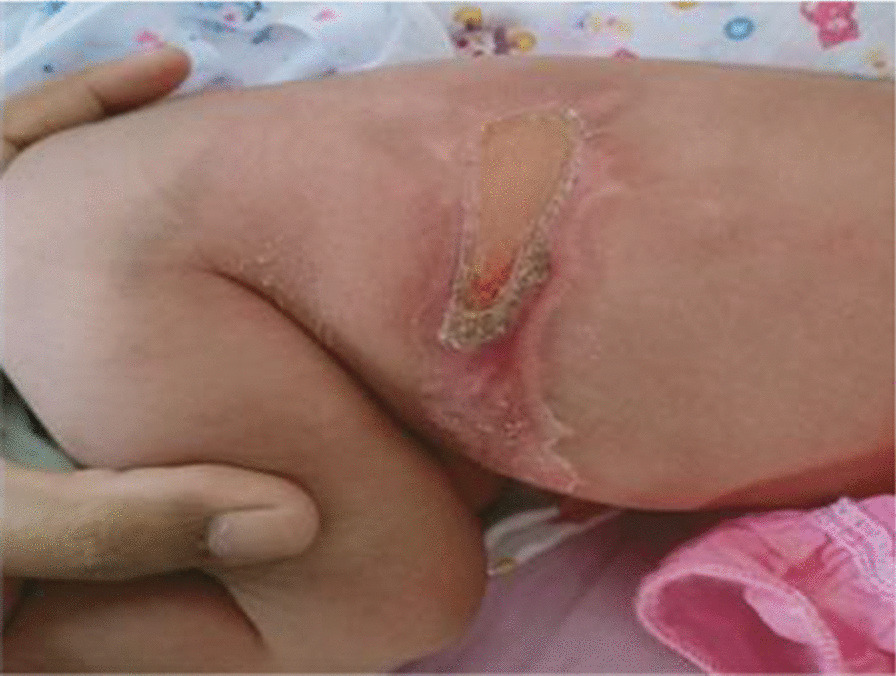
Aplasia cutis distributed over left thigh

Physical examination revealed a well-looking, active, alert, and pink neonate. Length was 52 cm, and head circumference was 34 cm. Neonatal examination was suggestive of six skin defects compatible with multiple aplasia cutis congenita. Two defects measured 10 cm in diameter and were distributed over right and left sides of abdomen (Fig. 1). Two additional skin defects were noticed on her head (Fig. 2). There were also two more healed lesions distributed over left thigh, suggestive of possible intrauterine healing of cutis aplasia (Fig. 3). There were no associated limb abnormalities or skin rashes. The rest of the neonatal examination was unremarkable.

Ultrasounds of abdomen and brain were normal. Echocardiography also revealed structurally normal heart. Skeletal survey was normal. Hearing and ophthalmological examination were normal. Inflammatory markers and blood counts were normal, with no evidence of infection. Chromosomal study revealed a normal female baby with no chromosomal abnormalities. Histology of skin defects was not performed as parents did not consent to the procedure.

The child was commenced on antibacterial cream for skin defects. Review at 4 weeks of age showed evidence of complete recovery, leaving six atrophic scars on the body. Weight gain was appropriate for age (700 g over first 4 weeks).

Mother was reassured regarding the isolated nature of skin defects and absence of underlying abnormalities. She was referred to plastic surgeon for management of scars and follow-up. The last review at 3 months revealed normal development and physical growth.

## Discussion

ACC is a rare group of heterogeneous disorders [13] that are associated with either failure of the skin to fully develop or skin degeneration [14]. Although the exact mechanism is not fully understood, many risk factors have been implicated, including chromosomal abnormalities, intrauterine trauma, and amniotic defects [6]. Frieden [14] proposed a classification system for ACC consisting of nine groups based on the number and location of lesions and the presence or absence of associated malformations (Table 1). As this neonate had two scalp lesions with four other lesions on the body without other complications and screening for involvement of other systems such as heart, brain, ear, eye, and abdominal organs was normal, the most likely diagnosis would be type V ACC. This diagnosis was further supported by fetus papyraceous confirmed with the demise of second twin during antenatal period.

Type 5 aplasia cutis is associated with multiparous pregnancy, abnormal placental vascular anastomosis leading to placental infarctions, and fetus papyraceous [15]. It has been reported in both monozygotic and dizygotic twin pregnancies with fetus papyraceous [16]. It is believed that the surviving twin can be affected by either hypotension or thrombotic events that occur secondary to demise of the other twin [17]. A recent literature review reported that mean gestational age of fetal demise is 13.3 weeks, and no gender predisposition was noted in children affected with type V aplasia cutis [18].

Aplasia cutis in the surviving twin can be suggested ultrasonically during the antenatal period by detection of high amniotic fluid index and relatively small abdominal wall circumference, and biochemically by elevated maternal alpha-fetoprotein levels [17]. The distribution of cutis aplasia is said to be bilaterally symmetrical and H-shaped over abdomen, buttock, and thighs in neonates with type V defects [18]. This characteristic involvement is likely related to distribution of watershed areas that are more likely to undergo ischemia-induced damage. However, literature suggests that clinical presentation, localization, extension, and extracutaneous involvement can be heterogeneous [19]. Similarly, in this child, in addition to typical distribution of cutis aplasia defects, additional defects were found on scalp.

The diagnosis of type V aplasia cutis is made on clinical grounds which support characteristic distribution and background fetus papyraceous. However, the diagnosis can be supported by demonstration of antenatal radiological and biochemical abnormalities, biopsy of skin defects to demonstrate absence of epidermis, dermis, and subcutaneous fat, and placental histological abnormalities.

Short-term complications of type aplasia cutis include infection, desiccation, and electrolyte imbalances. Therefore, it is recommended that extensive defects be cared for similar to burn injuries by application of local antimicrobial agents, and silver sulfadiazine on exposed areas and that the defects then be covered with petroleum gauze, dry gauze, and self-adherent wraps [20]. Contractures can be formed as long-term sequelae during healing process, and rigorous massage may help avoiding surgical release of these contractures [20]. Physiotherapy is indicated to prevent contractures related to restriction of limb movements. In children with extensive aplasia cutis, surgical grafting may be indicated [21]. Since this reported newborn had multiple superficial lesions only, she responded well to local antibiotic creams and conservative management.

**Table 1:** Frieden’s classification of aplasia cutis congenita

1. ACC located on the scalp with no other anomalies
2. ACC located on the scalp but with concomitant limb anomalies such as: limb malformations (Adams–Oliver syndrome), hypoplasia or aplasia of the distal phalanges, vascular malformations, fibromas, nipple and hair abnormalities
3. ACC of the scalp along with epidermal nevi, neurological, and ophthalmic abnormalities (such as seizures, mental impairment, corneal and eyelid lesions)
4. ACC accompanied by embryologic deformities: such as omphalocele, leptomeningeal angiomatosis, cranial stenosis, porencephaly, meningomyelocele, spinal dysraphism, or gastroschisis
5. ACC along with fetus papyraceous, placental infarct; extensive ACC of the trunk or limbs
6. ACC and epidermolysis bullosa involving the lower extremities
7. ACC with no epidermolysis bullosa involving the extremities
8. Teratogen-associated ACC: herpes simplex and varicella-zoster virus intrauterine infections, and drugs during pregnancy such as methimazole or carbimazole
9. ACC accompanied by congenital malformations [Patau syndrome (trisomy 13), Wolf–Hirschhorn (4p deletion), Setleis syndrome, Johanson–Blizzard syndrome, Goltz syndrome, ADAM complex, Kabuki syndrome, Delleman syndrome, Finlay–Mark syndrome, XY gonadal dysgenesis]

## Conclusions

Type 5 aplasia cutis congenita is a rare condition with unknown etiology. The diagnosis is made on clinical grounds which support characteristic distribution and background fetus papyraceous. Management depends on several factors including its pattern, location, severity, underlying causes, and associated anomalies. Superficial lesions are managed conservatively, whilst extensive lesions may warrant surgical grafting.

## Data Availability

The data that support the findings of this case report are available from Medical Records Department, Batticaloa Teaching Hospital, but restrictions apply to the availability of these data, which were used under license for the current report and so are not publicly available. Data are, however, available from the authors upon reasonable request and with permission of Medical Records Department, Batticaloa Teaching Hospital, Sri Lanka.

## Abbreviation

ACC: Aplasia cutis congenita
